# Internal Clocks, mGluR7 and Microtubules: A Primer for the Molecular Encoding of Target Durations in Cerebellar Purkinje Cells and Striatal Medium Spiny Neurons

**DOI:** 10.3389/fnmol.2019.00321

**Published:** 2020-01-10

**Authors:** S. Aryana Yousefzadeh, Germund Hesslow, Gleb P. Shumyatsky, Warren H. Meck

**Affiliations:** ^1^Department of Psychology and Neuroscience, Duke University, Durham, NC, United States; ^2^Department of Experimental Medical Science, Lund University, Lund, Sweden; ^3^Department of Genetics, Rutgers University, Piscataway, NJ, United States

**Keywords:** interval timing, temporal memory, cerebellum, striatum, microtubule dynamics

## Abstract

The majority of studies in the field of timing and time perception have generally focused on sub- and supra-second time scales, specific behavioral processes, and/or discrete neuronal circuits. In an attempt to find common elements of interval timing from a broader perspective, we review the literature and highlight the need for cell and molecular studies that can delineate the neural mechanisms underlying temporal processing. Moreover, given the recent attention to the function of microtubule proteins and their potential contributions to learning and memory consolidation/re-consolidation, we propose that these proteins play key roles in coding temporal information in cerebellar Purkinje cells (PCs) and striatal medium spiny neurons (MSNs). The presence of microtubules at relevant neuronal sites, as well as their adaptability, dynamic structure, and longevity, makes them a suitable candidate for neural plasticity at both intra- and inter-cellular levels. As a consequence, microtubules appear capable of maintaining a temporal code or engram and thereby regulate the firing patterns of PCs and MSNs known to be involved in interval timing. This proposed mechanism would control the storage of temporal information triggered by postsynaptic activation of mGluR7. This, in turn, leads to alterations in microtubule dynamics through a “read-write” memory process involving alterations in microtubule dynamics and their hexagonal lattice structures involved in the molecular basis of temporal memory.

## Introduction

Studies of time perception and timed performance for sub- and supra-second durations have frequently highlighted the involvement of cerebellar Purkinje cells (PCs) in the absolute timing of single sub-second durations. In contrast, striatal MSNs are thought to be involved primarily in the relative timing of beat-based sequences (e.g., Lusk et al., 2016; for additional details, see Grube et al., 2010; Teki et al., 2011, 2012; Breska and Ivry, 2016, 2018). Although these networks have been designed to function in a coordinated manner in order to maximize the accuracy and precision of timed behavior across their full range of coverage, they are more typically studied separately by behavioral and cognitive neuroscientists given the degree of specialization required to disentangle the complex behavioral profiles and underlying neural circuitry. As a consequence, temporal processing in the cerebellum and basal ganglia are only now being widely integrated into a coherent timing system (e.g., Teki et al., 2012; Bostan et al., 2013; Petter et al., 2016; Bareš et al., 2019; Caligiore et al., 2019).

Numerous circuit diagrams have been developed that specify the primary “time cells” and their relation to the clock, memory, and decision stages of temporal processing, as well as the extent and locations of plasticity in the brain areas where temporal learning and memory can co-occur. Interestingly, although virtually all timing models make an attempt to explain how time is measured and utilized (see Matell and Meck, 2000; Hass and Durstewitz, 2016; Matthews and Meck, 2016) few of them provide specific details for how target durations are encoded and decoded within the proposed neural circuitry (Merchant and de Lafuente, 2014). This means that the form and content of temporal memory, or whether it is written and read at a single-cell level or across a distributed network is typically left unaddressed (Meck, 1983, 2002; Teki et al., 2017; Paton and Buonomano, 2018).

Recent experimental evidence suggests that the metabotropic glutamate receptor 7 (mGluR7) regulates glutamate-mediated postsynaptic inhibition in cerebellar PCs (e.g., Phillips et al., 1998; Johansson et al., 2015) and striatal medium spiny neurons (MSNs; e.g., Borroto-Escuela et al., 2018; Briones et al., 2018), thereby activating a biochemical-signaling cascade that results in an intracellular form of temporal memory. In an attempt to determine the relevancy of other components of these signaling cascades in PCs and MSNs, we review the literature with the goal of promoting further consideration of the potential functions that cytoskeletal elements, such as microtubules, are able to provide due to having the structural complexity, integrity, and longevity necessary to support the sophistication of read-write memory processes and temporal cognition.

Neural memory mechanisms are typically considered to be responsible for maintaining a continuum of events in a *retrievable* form. In this manner, read-write memory processes allow neural networks to “recode past information in light of current information” (Gallistel and Wilkes, 2016, p.12). Although there’s been a veritable explosion in the last few decades about our understanding of the complementary roles of long-term potentiation (LTP) and long-term depression (LTD) in terms of the strengthening and weakening of synapses involved in learning and memory, there are still huge gaps in our understanding of how memories are stored in the brain. These gaps are such that there are even uncertainties as to whether LTP plays a critical role in memory storage or is perhaps better viewed as an enhanced form of attention that leads to sustained information processing to support encoding and decoding of memories (e.g., Shors and Matzel, 1997; Gallistel and Balsam, 2014; Trettenbrein, 2016; Gallistel, 2017; Queenan et al., 2017). Whether microtubules act as one of the elements in the molecular signaling cascades that regulate synaptic plasticity (e.g., studies discussed in Dent, 2017), or is the main substrate for storing intracellular memories (e.g., Hameroff et al., 2010), remains to be addressed. Either way, it is important to shed light on the importance of microtubules in the formation and consolidation of temporal information in future studies.

As suggested above, studies aimed at elucidating the behavioral and neural mechanisms that control the accuracy and precision of timed eye-blink responses became highly influential in the study of the representation of time for motor learning. This was due, in part, to how elegantly-designed and specific the cerebellar circuitry appeared to be (Medina et al., 2000). So much so that the well-known neuroanatomist *David Marr* suggested that cerebellum-dependent learning highly relies on the synaptic plasticity between parallel fibers and PCs (Marr, 1969):

*“The main test of the theory is whether or not the synapses from parallel fibers to PCs are [modifiable]…. It is likely that no other cerebellar synapses are modifiable…. Though it is difficult to see how these predictions could be wrong, they might be: such a disproof would be embarrassing but not catastrophic, since something of the bones of the theory would remain.”* (Marr, 1969; for additional details, see Carey and Lisberger, 2002; Cheron et al., 2013).

This over-reliance on the supposedly “hard-wired” or “reflexive nature” of the cerebellar circuitry was to such an extent that it was thought that only the PCs in the cerebellar cortex are capable of plasticity mechanisms and learning (e.g., Schneiderman and Gormezano, 1964; McCormick and Thompson, 1984; Krupa et al., 1993). It has recently been demonstrated that PCs acquire and maintain a temporal code independent from the LTD of parallel fibers connected to PC synapses or inhibitory inputs they receive from parallel fibers or GABAergic interneurons. Moreover, these observations have led to the proposal of an internalized temporal memory in PCs linked to an intrinsic cellular mechanism rather than a circuit-based pattern (Johansson et al., 2014, 2018). Based on these provocative results, PCs appear to be capable of learning temporal response patterns without receiving external temporal input which is a very challenging result for any theoretical account put forth this point, except perhaps for the recently proposed ICAT integrative timing model by Petter et al., 2016 (also see Jirenhed et al., 2017; Bareš et al., 2019). Using various glutamate receptor antagonists, it was determined that metabotropic glutamate receptor 7 (mGluR7) facilitates this internal temporal memory through glutamate-mediated postsynaptic inhibition, a process that has been previously reported in PCs (Inoue et al., 1992; Johansson et al., 2015). It has been proposed that mGluR7 activation in PCs initiates a biochemical-signaling cascade, that may support the encoding of the temporal components of the evoked conditioned response (Johansson et al., 2015, 2016). However, the components of this biochemical-signaling cascade are still largely unknown. Accordingly, the role mGluR7 in the acquisition and extinction of conditioned responses has been extensively studied, and it has been indicated that aversive learning and fear response is impaired in mGluR7 knock-out or deficient mice (e.g., Masugi et al., 1999; Cryan et al., 2003; Callaerts-Vegh et al., 2006; Goddyn et al., 2008) Moreover, mice show improved performance in a contextual fear conditioning task as a result of mGluR7 potentiation (Gogliotti et al., 2017).

## Cerebellar Timing Circuit

Prospective timing involves learning the absolute and relative durations of stimuli in order to anticipate the future occurrence of significant events. Classical conditioning of the eyeblink reflex is a commonly used task to study the acquisition and retention of the temporal relation(s) between a conditioned stimulus (CS) and an unconditioned stimulus (US). In order to avoid the delivery of an aversive US to the eye (e.g., air puff) the subject learns to utilize this temporal information in order to make an anticipatory eyeblink, thereby minimizing the negative impact of the US (e.g., Hesslow and Ivarsson, 1994; Christian and Thompson, 2003; Johansson et al., 2018). Although the cerebellum has typically been viewed as being specialized for the timing of sub-second durations in the control of movement, a number of recent reports have also implicated a role in the timing of supra-second durations in support of cognition (e.g., Ohmae et al., 2017; Kunimatsu et al., 2018). These studies emphasize the anatomical projections from the cerebellar cortex to the dentate nucleus (DN), and then beyond the cerebellum to thalamocortical-striatal circuits. The current hypothesis is that the cerebellum is not strictly limited to temporal processing in the millisecond range, but also plays an important role in temporal processing in the hundredths of milliseconds-to-minutes range as a result of its coordination with cortico-striatal circuits (Merchant et al., 2013; Petter et al., 2016).

### Purkinje Cells

PCs are the primary cell type found throughout the cerebellar cortex. PCs in the lateral cerebellar cortex are considered crucial for predicting the temporal relation(s) between the CS and US in eyeblink conditioning. Evidence for the role of PCs in temporal prediction comes from the observation of conditioned and adaptively timed pauses in spiking activity, which are acquired as a function of repeated pairings of the CS and US (Jirenhed et al., 2007; Jirenhed and Hesslow, 2011). Moreover, these pauses in neural spiking responses have been shown to be sufficient for eliciting changes in the temporal control of motor responses such as eyeblinks (Heiney et al., 2014). Later findings suggest that PCs do not simply receive timing input from upstream neural circuits, but rather, that the timing of anticipatory responses by the cerebellum is intrinsic to PCs controlling the duration of pauses in firing activity (Johansson et al., 2016, 2018). Overall, these observations support the hypothesis that an intrinsic memory process in PCs encodes the details necessary for the temporal control of eyeblink conditioning in a manner that is co-occurring with and independent of the neural mechanisms typically thought to support associative learning (e.g., LTP and LTD—see Wilkes and Gallistel, 2017 for a discussion of information theory and the role of interval timing in associative learning).

### Deep Cerebellar Nuclei

The temporal information encoded in PCs allowing for the control of the duration of pauses in firing is propagated downstream until it reaches the deep cerebellar nuclei. Because PC action potentials are regulated by GABA, these timed pauses in PC spiking activity produce a form of disinhibition in these deep cerebellar nuclei. Correlations between neural ramping activity and eye movements have been observed in the DN of monkeys performing a timed ballistic eye-movement task for intervals in both sub- and supra-second ranges (Ohmae et al., 2017).

Other lines of evidence suggest that the outputs from deep cerebellar nuclei contribute to the fine-tuning of predictive timing in both milli-second and multi-second time ranges in humans, monkeys, and rats through the adjustment of downstream neural circuits (e.g., Callu et al., 2009; Ohmae et al., 2013, 2017; Broersen et al., 2016; Parker, 2016). The relevant pathways include efferent outputs from the DN in primates or the lateral cerebellar nucleus (LCN) in rodents, both of which exhibit di-synaptic connections, *via* the thalamus, to the cortico-striatal circuits that sub-serve timing in the seconds-to-minutes range (e.g., Coull et al., 2011; Bostan et al., 2013; Merchant et al., 2013). Importantly, stimulation of thalamic terminals has been observed to facilitate LCN output to these cortico-striatal circuits and enhance the precision of multi-second timing (Parker et al., 2017). Furthermore, performance in predictive timing tasks synchronizes ramping activity and theta-frequency oscillations in the frontal cortex and cerebellum (Parker, 2016). It is important to note, however, that although “ramping” may provide a useful description of the neural activity observed during temporal processing, it is likely that computational models incorporating stepping dynamics offer a more complete account of the underlying timing mechanism(s) than models utilizing ramping dynamics (e.g., Latimer et al., 2015, 2017). Moreover, as cautioned by Kononowicz et al. (2018) and Paton and Buonomano (2018), ramping activity (in contrast to population clocks) is often best described as representing activity in a brain area that is monitoring some unknown time signal occurring elsewhere in the brain, rather than the locus of a clock, i.e., generator of the time base.

## Striatal Timing Circuit

### Medium Spiny Neurons

MSNs are the dominant cell type within the dorsal striatum, which receive extensive glutamatergic input from cortical areas and thalamus, as well as dopaminergic input from the ventral tegmental area (VTA) and substantia nigra pars compacta (SNc; Cheng et al., 2006, 2007; Huerta-Ocampo et al., 2014; Agostino and Cheng, 2016). Using two-photon microscopy, and two-photon glutamate uncaging to examine sub-threshold synaptic integration in MSNs, Carter et al. (2007) observed that synaptic responses can summate sub-linearly, linearly, or supra-linearly depending on the spatiotemporal pattern of activity. Importantly, synaptic responses modulated by N-methyl-D-aspartic acid receptors (NMDARs), which are important for the induction of synaptic plasticity, summated linearly as a function of repetitive activity (Carter et al., 2007). Consequently, sub-threshold integration of electrical potentials in MSNs is influenced by the arrangement of synaptic inputs and the differential firing patterns of multiple postsynaptic neurons with oscillatory properties while asynchronous synaptic inputs to neighboring spines do not interact (Carter et al., 2007). Overall, MSNs demonstrate an ability to support high levels of integration at multiple modes as determined by the spatial and temporal distributions of their synaptic inputs and outputs. This makes them well-suited as temporal integrators for the linear time dimension specified by the scalar timing theory (Gibbon et al., 1984, 1997; Matell et al., 2003).

### Computational Properties of the Striatum

The main feature that makes cortico-striatal circuits well-suited for accurate and precise timing, is the high level of connectivity between the MSNs *via* GABAergic interneurons, which results in coordinated activation of MSN populations and a high signal to noise ratio (Moyer et al., 2014). Moreover, dopamine-mediated synaptic plasticity and spike-timing-dependent plasticity in this region can greatly facilitate value assignment and learning of target durations and timed behavior (e.g., Shen et al., 2008; Xu and Baker, 2016). There are several reports indicating that the glutamatergic input and the phasic dopamine bursts in the striatum need to be paired with each other and occur in a very precise temporal window for plasticity to happen (Yagishita et al., 2014; Wieland et al., 2015). It is important to note that these findings are most often obtained from MSNs in the ventral striatum which although very similar to MSNs in the dorsal striatum, are considered to be more involved in reward than in temporal processing. Despite this potential confound, the current view is that mechanisms of neural plasticity do not differ between the MSNs in the ventral and dorsal striatum.

Although a comprehensive understanding of the cellular and molecular basis of interval timing awaits further investigation, the development of the striatal beat-frequency (SBF) model of interval timing (Matell and Meck, 2000, 2004) continues to serve as an important guidepost for directing future research (e.g., Farrell, 2011; Oprisan and Buhusi, 2011; Soares et al., 2016; Teki, 2016; Dallérac et al., 2017; Toda et al., 2017; Gu et al., 2018). The main reason for this is that the SBF model provides a neurobiologically plausible account of interval timing within cortico-striatal circuits that can be extended and revised as additional information becomes available. At present, its core feature is the reliance on coincidence detection of oscillatory inputs from the cortex and thalamus by MSNs in the dorsal striatum. MSNs are trained over successive trials by synaptic plasticity mechanisms to function as detectors of unique patterns of input that are related to specific target durations paired with reinforcement (Dallérac et al., 2017). A major strength of the model is that it accounts for the scalar property which is the hallmark of interval timing (Allman et al., 2014; Yin et al., 2017). The first component of the scalar property requires that the mean measures of the timed behavior vary linearly, and usually accurately, with imposed temporal standards (i.e., target durations). The second component is the scalar property of variance, a form of Weber’s law, which requires timing sensitivity to remain constant as the target durations being timed vary. Timing variance can be evaluated by taking the standard deviation (σ - “sigma”) and the mean (μ - “mu”) of the timing behavior for various target durations. This allows one to calculate the coefficient of variation (CV) where CV = σ/μ, a Weber-fraction measure. Consequently, the scalar property of variance asserts that variations in the target duration do not alter the CV. Conventionally, the scalar property of timing is typically stated as the variability growing proportional to the mean of the target duration(s) being timed (Gibbon et al., 1984, 1997). The SBF model also accounts well for the anatomical, pharmacological, and electrophysiological properties of interval timing (Buhusi and Meck, 2005; Meck, 2006; Balci et al., 2008; Coull et al., 2011; Merchant et al., 2013; Gu et al., 2015, 2018; Toda et al., 2017).

## Integrative Models of Temporal Processing

### Initiation, Continuation, Adjustment, and Termination

**Figure 1:**
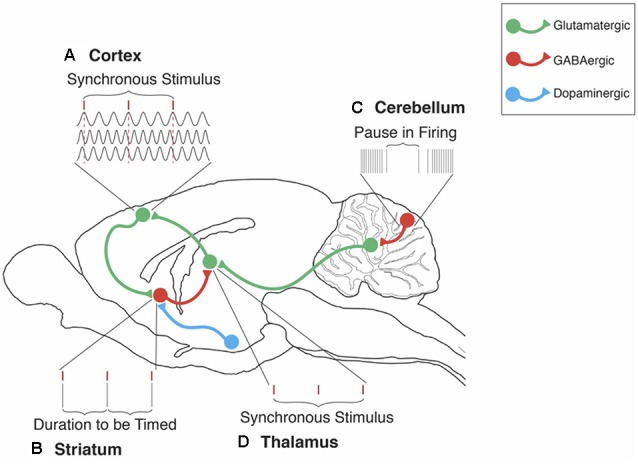
The cerebellum’s contribution to the striatal beat frequency (SBF) model of interval timing. **(A)** Cortical neurons oscillate at different frequencies (typically distributed between 5 and 15 Hz). These oscillations are synchronized by phasic dopamine (DA) release mostly from the ventral tegmental area (VTA). Each tick mark represents the peak excitatory phase of an oscillatory input onto a medium spiny neuron (MSN). **(B)** At the duration to be timed, cortical-striatal synapses that are active, as indicated by a peak phase of oscillatory input, will experience long-term potentiation (LTP) due to the presence of DA. **(C)** Cerebellar Purkinje cells (PC) are conditioned to pause firing at trained durations in order to produce accurately timed anticipatory responses. These pauses in PC firing regulate neural activity primarily in the dentate nucleus (DN). **(D)** Disinhibition of the DN then proceeds to the thalamus, thus supporting the regulation of timing in cortical-striatal circuits. In this manner, the ICAT model provides an integrated framework for integrating cerebellar and striatal support for interval timing in both sub and supra-second ranges. In this model, Initiation refers to the “start” of interval timing signaled by the onset of endogenous or exogenous stimuli. Initiation deficits contribute to decreased accuracy in the temporal control of behavior. Continuation refers to the maintenance of the internal timing process during the target duration. Adjustment refers to the real-time monitoring and fine-tuning of the internal timing process in order to increase precision through feedback and error correction. Termination refers to the “stop” of internal timing following the cessation of the to-be-timed stimulus. Adapted from Petter et al. (2016) and Bareš et al. (2019).

The contributions of PCs and deep cerebellar nuclei to the timing of sub- and supra-second durations have been incorporated into the Initiation, Continuation, Adjustment, and Termination (ICAT) model of temporal processing (Petter et al., 2016; see also Bareš et al., 2019; Caligiore et al., 2019). The ICAT model was explicitly designed to support real-time interaction between cerebellar and cortico-striatal circuits during sequential phases of predictive timing for durations in the of milliseconds-to-minutes range as diagrammed in Figure 1. The cellular architecture of the cerebellum, with its parallel fibers bifurcating to form T-shaped branches that provide temporal and motor learning information to PCs through excitatory synapses, makes it ideal for supporting the *initiation* and *adjustment* phases of the ICAT model with its major impact being observed in the timing of discrete intervals as opposed to continuous cyclic intervals (e.g., Spencer et al., 2003, 2005; Breska and Ivry, 2016). The ICAT model also accounts for the central role that the cerebellum plays in the automatic timing of reflexive motor behaviors (e.g., Rasmussen and Jirenhed, 2017). In contrast, cortico-thalamo-striatal circuits provide more cognitively controlled regulation of the *continuation* phase for the timing of both discrete and continuous cyclic movements (Merchant and Yarrow, 2016). The ICAT model is also congruent with clinical observations of motor and timing deficits exhibited by patients with cerebellar dysfunction (e.g., Schmahmann, 2004; Bares et al., 2011; Lungu et al., 2016).

While the cerebellum has traditionally been studied in terms of its role in motor control and the timing of movements (e.g., saccades), it is now becoming recognized as contributing to a broader range of temporal processes involved in attention, language, and other types of cognition (Buckner, 2013). Although adaptive pauses in PC spiking are sufficient for timing sub-second intervals, cerebellar integration with cortical-striatal circuits is necessary for timing supra-second intervals. In this instance, the cerebellum monitors the timing of supra-second intervals and provides error-detection for fine-tuning the signal in cortico-striatal circuits. Consequently, the cerebellum’s role in temporal processing should be viewed within the context of a global timing network that includes cerebellar-cortical, cortico-striatal, and the hippocampal cortical circuits (e.g., Meck et al., 2013; MacDonald et al., 2014; Lungu et al., 2016; Lusk et al., 2016; Petter et al., 2016, 2018; Raghavan et al., 2016; Bareš et al., 2019; Caligiore et al., 2019).

## Molecular Mechanisms of Temporal Memory

### Potential Role(s) of Microtubules and mGluR7 in Temporal Memory

As an additional element to the previous findings regarding temporal processing mechanisms in PCs, we propose a specific role for microtubules in the molecular mechanisms that follow mGluR7 activation in PCs. Microtubules are dynamic cytoskeletal structures composed of αβ-tubulin heterodimers. They are involved in synapse-to-cell-body trafficking, chromosome segregation, and morphogenesis (Aher and Akhmanova, 2018). Numerous empirical studies have identified a relationship between microtubules and memory processes (e.g., Fanara et al., 2010; Barten et al., 2012; Dent and Baas, 2014; Uchida et al., 2014; Atarod et al., 2015; Smythies, 2015; Uchida and Shumyatsky, 2015, 2018; Martel et al., 2016; Dent, 2017; Yousefzadeh et al., in press). Additionally, various characteristics of Alzheimer’s disease (AD) are correlated with changes in the structure and dynamics of microtubules through both tau-dependent and tau-independent mechanisms (Brandt and Bakota, 2017).

It has been demonstrated that α-tubulin directly interacts with mGluR7 (Saugstad et al., 2002), and mGluR7 activation leads to the activation of the mitogen-activated protein kinase pathway (MAPK) leading to microtubule stabilization (Jiang et al., 2006; Gu et al., 2012, 2014). Consequently, we propose that postsynaptic activation of mGluR7 leads to alterations in microtubule dynamics, which could transiently inhibit PC firings. Thus, PCs are capable of maintaining a temporal code through the time-specific pauses that occur in their spike patterns in the millisecond range. A similar process occurring in striatal MSNs would allow for changes in the speed of temporal integration and the coincidence detection of specific target durations in the seconds-to-minutes range. With all of this taken into account, it is realistic to consider the possibility that microtubules play a key role in the biochemical-signaling cascade that encodes the durations of events in single cells on individual trials, thus providing PCs and MSNs the possibility of serving as independent “time cells.” This provides a unique opportunity for investigators to search for the temporal engram and whether there are different varieties of these time cells even in the same brain structure. As *Randy Gallistel* has so aptly put it on more than one occasion, “It is much easier to formulate a coding hypothesis if the engram is realized by a cell-intrinsic molecular mechanism” (Gallistel, 2017, p. 498).

#### Microtubules in Dendritic Spines

Microtubules are one of the most fundamental elements in establishing the structure and function of neurons. The intrinsic polarity and dynamic structure of microtubules makes them suitable for organizing neuronal morphogenesis, including neural migration, neuritogenesis, neurite outgrowth, branching, and retraction (Fukushima, 2011; Baas et al., 2016). Moreover, microtubules are involved in axonal and dendritic cargo transport (Maday et al., 2014; Hirokawa and Tanaka, 2015), spike transduction (Friesen et al., 2015), and synapse modulation (Jaworski et al., 2009). Although microtubules are far more abundant in the cell bodies and along the axons of neurons, the first location they interact with receptors and ion channels is at dendritic spines (Gardiner et al., 2011). Originally, it was believed that the only cytoskeletal elements available in dendritic spines were actin filaments, whereas microtubules and microtubule-associated proteins (MAPs) are only present in dendritic shafts, and do not enter dendritic spines (Kaech et al., 2001). Studies conducted by Gu et al. (2008), Hu et al. (2008), Mitsuyama et al. (2008) and Jaworski et al. (2009) however, challenged this notion. It was shown that brain-derived neurotrophic factor (BDNF), a molecule critically involved in learning and memory, induces the entry of dynamic microtubules into dendritic spines as a function of neuronal firing (Hu et al., 2008). Through interaction with actin filaments, these dynamic microtubules contribute to regulating the morphology of dendritic spines and, as a consequence, synaptic plasticity (Jaworski et al., 2009; Coles and Bradke, 2015; Peris et al., 2018). Formation of mushroom-shaped spines and spine enlargement happens as a result of dynamic microtubule entry, both of which accelerate synaptic strength (Hoogenraad and Bradke, 2009; Kapitein and Hoogenraad, 2015). Moreover, dynamic microtubule entry into synaptic spines contributes to NMDAR-dependent synaptic plasticity (Kapitein et al., 2011; Merriam et al., 2011).

There have also been reports of learning-induced changes in microtubule dynamics that are regulated by the phosphorylation status of stathmin, a microtubule-destabilizing phosphoprotein. At synaptic sites in the dentate gyrus of the hippocampus, stathmin undergoes steps of dephosphorylated and phosphorylated states causing biphasic shifts in microtubule dynamics, which modulates AMPA receptor trafficking (Kim and Lisman, 2001; Uchida et al., 2014; Uchida and Shumyatsky, 2015; Kaganovsky and Wang, 2016; Martel et al., 2016). Stathmin phosphorylation also regulates dendritic arborization in cerebellar PCs and its overexpression in these cells leads to motor discoordination (Ohkawa et al., 2007a,b). Moreover, significant correlations among irregularities in stathmin, microtubule dynamics, and memory impairments have been observed in aged animals (Uchida et al., 2014). Similarly, stathmin deficient mice exhibit impairments in spike-timing-dependent plasticity in the lateral amygdala which is associated with deficiencies in parental and social behavior as well as in recognizing innate and learned fear (Shumyatsky et al., 2005; Martel et al., 2008). Other studies have demonstrated a need for this regulatory protein in the maintenance of axonal microtubules (Duncan et al., 2013).

As described by Gardiner et al. (2011), there appears to be a bidirectional interaction between neurotransmitters and microtubules. On one hand, neurotransmitters are capable of activating signaling pathways that regulate microtubule dynamics or expression levels. They are also involved in enforcing various post-translational modifications on tubulins. On the other hand, microtubules can influence receptor concentration in the postsynaptic neuron. They can also facilitate electrical current transduction, leading to the regulation of neurotransmission (Gardiner et al., 2011).

#### Microtubules and mGluR7

As mentioned earlier, mGluR7 is a G-protein coupled receptor, and one of the members of type III metabotropic glutamate receptors. This protein has been attributed to cognitive functions including learning, memory, and emotion regulation. Although mGluR7 typically operates as a pre-synaptic auto-receptor and constrains glutamate release from pre-synaptic terminals, there are reports of post-synaptic mGluR7 functions as well (for review, see Palazzo et al., 2016; Tassin et al., 2016). Studies on the regulatory effects of postsynaptic mGluR7 on NMDA receptors have indicated that mGluR7 activates the MAPK pathway in the basal forebrain cholinergic neurons and prefrontal cortex pyramidal neurons, which in turn causes increased cofilin activity and actin depolymerization (Gu et al., 2012, 2014). The MAPK signaling pathway has been implicated in synaptic plasticity mechanisms and thereby learning and memory (for a review of the MAPK pathway, see Thomas and Huganir, 2004). Studies have indicated that MAP kinases are colocalized with microtubules in neuronal processes (Fiore et al., 1993) and regulate microtubule dynamics (Reszka et al., 1995; Pei et al., 2002; Pullikuth and Catling, 2007). Such negative regulation of the MAPK pathway results in the activation of stathmin, which promotes microtubule destabilization (Jeanneteau et al., 2010). Other than the MAPK pathway, mGluR7 also acts through other signaling cascades, including the inhibition of cAMP-dependent pathway and the modulation of PI-3-K pathway (Iacovelli et al., 2002, 2014). Moreover, mGluR7 directly interacts with α-tubulin *via* its C-terminus domain (Saugstad et al., 2002). This dynamic interaction is negatively regulated by receptor activation, i.e., mGluR7 activation reduces its binding affinity for α-tubulin. Because the C-terminus domain of this receptor binds to many other regulatory molecules (including calmodulin, PICK1, PKC, etc.), the interaction between mGluR7 and α-tubulin can be involved in controlling how these regulatory molecules access mGluR7, thereby directing signal transduction mechanisms (Saugstad et al., 2002). Although the specifics on the function of mGluR7/α-tubulin interaction are not as yet determined, Jiang et al. (2006) explicated an association between type III mGluRs and Parkinson’s disease (PD). The findings from this article demonstrated that type III mGluR activation is able to attenuate the toxic effects of rotenone on dopaminergic neurons through the MAPK pathway activation, and thus microtubule stabilization. Using type III mGluR agonists, MAPK pathway was activated, resulting in microtubule stabilization, which hindered the effects of rotenone on dopaminergic neurons and ameliorated the PD-like symptoms (Jiang et al., 2006). Putting the studies mentioned above together, it is possible to propose a similar mechanism that accounts for encoding an internal temporal memory in PCs, i.e., mGluR7 activation modulates microtubule dynamics through the MAPK pathway, thereby promoting time-specific pauses in PC firing.

## Conclusions

We now know that microtubule destabilization leads to impairments in neurogenesis, spinogenesis, the acquisition and retrieval of contextual fear memory, and learning-induced CREB-mediated gene transcription (Fanara et al., 2010; Martel et al., 2016). Moreover, microtubule dynamics contribute to the formation of spatial and object memory in rats (Yousefzadeh et al., in press). Meanwhile, paclitaxel-induced microtubule hyperacetylation and stabilization induces learning and memory deficits in rats (Dowdy et al., 2006; You et al., 2018). Emphasizing the importance of microtubule dynamics in memory encoding, microtubule’s initial instability immediately following training and microtubule’s hyperstability about 8 h later appears to be a key component of memory consolidation: in a contextual fear conditioning paradigm in mice, paclitaxel injected in the dentate gyrus immediately after training inhibited memory formation, but the same drug-enhanced memory when injected 8 h after training (Uchida et al., 2014). All of these studies support the notion that microtubule dynamics equilibrium is directly involved in learning and memory processes. Moreover, using molecular mechanics modeling and electrostatic profiling, Craddock et al. (2014) suggested that information is encoded in microtubules through their interaction with calcium-calmodulin dependent kinase II. This interaction modulates a specific phosphorylation pattern on microtubules and stathmin which can be considered “neural plasticity at the molecular level” (Craddock et al., 2012). The microtubule code originated from post-translational modifications and expression of diverse α- and β-tubulin isoforms regulates synaptic transmission and neural plasticity, and as a result facilitates memory formation and maintenance (Janke and Kneussel, 2010; Gadadhar et al., 2017; Magiera et al., 2018a; Magiera et al., 2018b).

**Figure 2:**
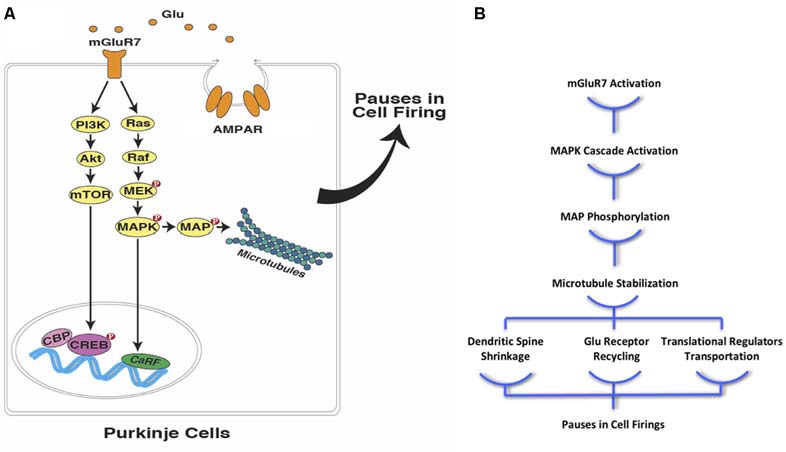
Interval timing at the sub-cellular level in PCs **(A)** mGluR7 mediated plasticity in cerebellar PCs is facilitated through the activation of mitogen-activated protein kinase pathway (MAPK) and PI-3-k pathways. These signaling cascades, directly and indirectly, lead to microtubule stabilization and contribute to precisely timed pauses in PCs firings in various ways including down-regulation of AMPA receptor distribution. **(B)** Moreover, microtubule stabilization as a result of postsynaptic mGluR7 activation in PCs can cause spine shrinkage and synapse-to-nucleus transcription regulator transport (Glu, glutamate; mGluR7, metabotropic glutamate receptor 7; AMPAR, AMPA receptor; NMDAR, NMDA receptor; PI3K, phosphoinositide 3-kinase; Akt, protein kinase B; mTOR, mechanistic target of rapamycin; MEK, MAPK/ERK Kinase; MAPK, mitogen-activated protein kinase; MAP, microtubule-associated protein; CBP, CREB-binding protein; CREB, cAMP response element-binding protein; CaRF, calcium responsive transcription factor; Red filled circles with P inside indicate protein phosphorylation).

Considering the well-established role of microtubules in hippocampal memory encoding (e.g., Woolf et al., 1999; Cavallaro et al., 2002; Uchida et al., 2014), it seems reasonable to assume that these cytoskeletal structures are also involved in the formation of a “temporal map” with instructions and/or an operators manual residing in cerebellar PCs. This temporal map plays a critical role in eye-blink conditioning, which influences PCs’ spike patterns. The pauses that normally occur in PC firings during the inter-stimulus interval (ISI), disinhibits the downstream neurons, triggering the conditioned eye-blink response. It has been shown that mGluR7 activation in postsynaptic PCs contributes to these spike patterns through the activation of biochemical signaling cascades that result in glutamate-mediated postsynaptic inhibition (Johansson et al., 2015). Since it has been shown that mGluR7 activates the MAPK signaling pathway and stabilize microtubules (Jiang et al., 2006; Jeanneteau et al., 2010; Gu et al., 2012, 2014), one can easily extend the same mechanism to temporal coding in PCs during eye-blink conditioning in rodents (Figure 2A). As a result of microtubule stabilization, the rate of dynamic microtubule entry into the dendritic spines of PCs declines, which leads to spine shrinkage, followed by LTD of the parallel fiber projections to the PCs. This process is similar to what has previously been described in the hippocampus due to NMDA receptor activation in postsynaptic neurons (Kapitein et al., 2011). This alteration in microtubule dynamics could further ameliorate synapse-to-nucleus transportation of synaptically-localized transcriptional regulators, such as CREB-mediated transcriptional coactivators (CRTC1), histone deacetylase 4 (HDAC4), and NF-κB, which are essential elements in synaptic plasticity (Figure 2B). The translocation of these synaptically-localized transcriptional regulators modulates gene transcription and advances memory formation, consolidation, and reconsolidation (e.g., Uchida and Shumyatsky, 2018; Yousefzadeh et al., in press).

Hameroff et al. (2010) proposed a neural basis for a long-sought form of “read-write” memory in the brain. Using molecular modeling, they were able to identify the spatial attributes of Ca^2^/calmodulin-dependent protein kinase II (CaMKII) domains that can accurately match those of microtubule hexagonal lattice neighborhoods, thus identifying potential phosphorylation mechanisms. The interaction between CaMKII and microtubules provides a testable framework for the molecular encoding of durations (Craddock et al., 2012). In this proposed interaction, tubulin dimers serve as memory bytes that can be written on by CAMKII, a protein that binds to these bytes and writes data on them. Dam1 would then serve as a “read” mechanism for the information encoded in the microtubule lattices. A primer for beginning this process might start with 6-tubulin memory bytes in the shape of blocks circling in a coil that makes up the microtubules. The idea for 6-tubulin memory blocks supports the proposal that Dam1 complexes could function as the “read” mechanism able to handle six or so blocks every step, and slide along the microtubule structure while rotating, reading from the lattice with every turn it does, a rotating array of read-heads. This mechanism considers microtubules as a substrate necessary for encoding information independent from mechanisms of neural plasticity, and supports proposals that do not rely on LTP/LTD as the major form of learning (e.g., Gallistel and Balsam, 2014).

One needs to keep in mind, however, that the types of molecular memory processes reviewed here are unlikely to be limited to any particular time range (e.g., sub- vs. supra-second timing—see Rammsayer and Troche, 2014). As a consequence, the emerging view in the field is that although the timing processes governed by cortical-striatal circuits are distinct from the timing processes governed by cortico-cerebellar circuits, there is considerable room for integration of behavioral, systems, cellular, and molecular mechanisms. As a consequence, the proposed ICAT model (Petter et al., 2016) assumes that these circuits work in synchrony, and contribute to distinct components of virtually all timing tasks (see Allman et al., 2014; Ohmae et al., 2017; Bareš et al., 2019; Caligiore et al., 2019).

**Figure 3:**
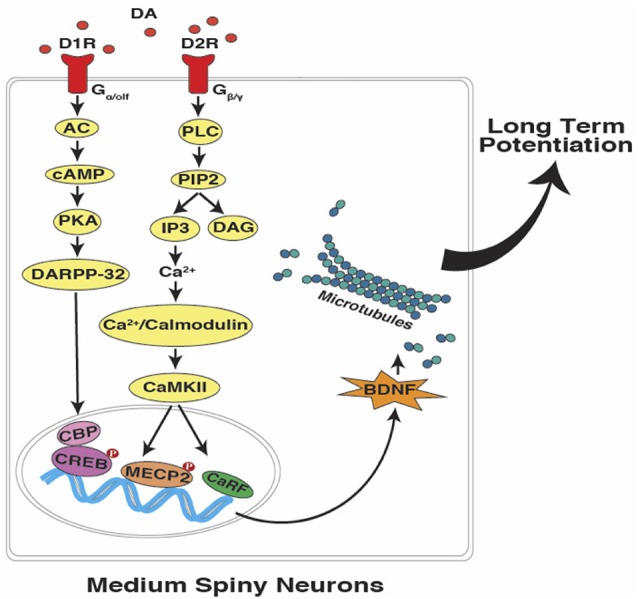
Interval timing at the sub-cellular level in striatal MSNs. In accordance with the SBF model of interval timing, dopamine-dependent LTP leads to the encoding of both sub and supra-second target durations. Activation of dopamine receptors can potentially lead to the up-regulation of brain-derived neurotrophic factor (BDNF), which promotes microtubule dynamics. Upon the entry of dynamic microtubules into the dendritic spines, bigger and mushroom-shaped spines emerge which support the acquisition and maintenance of LTP (DA, dopamine; D1R, D1-type receptor; D2R, D2-type receptor; AC, adenylyl cyclase; cAMP, cyclic adenosine monophosphate; PKA, protein kinase A; DARPP-32,dopamine- and cAMP-regulated neuronal phosphoprotein; PLC, phospholipase C; PIP2, phosphatidylinositol 4, 5-bisphosphate; IP3, inositol trisphosphate; DAG, diacylglycerol; CaMKII, Ca^2+^/calmodulin-dependent protein kinase II; CBP, CREB binding protein; CREB, cAMP response element-binding protein; MECP2, methyl CpG-binding protein 2; CaRF, calcium responsive transcription factor; Red filled circles with P inside indicate protein phosphorylation).

In summary, as described above, cerebellar PCs are capable of maintaining a temporal code through the time-specific pauses that occur in their millisecond spike patterns. A similar process, if verified in striatal MSNs would allow for changes in the speed of temporal integration and the coincidence detection of specific target durations in the seconds-to-minutes range (Figure 3). The proposal is that an intrinsic cellular mechanism based on microtubule dynamics (in both cerebellar and striatal “time cells”) encodes the relevant temporal information (e.g., target duration and response thresholds) and can possibly be more effective as a read-write memory system than a circuit-based system with anatomically distinct temporal processing stages (e.g., clock, memory, and decision) as outlined by Gallistel and King (2010), Allman et al. (2014) and van Rijn et al. (2014).

## Future Directions

Several lines of inquiry are required to further examine the cellular and molecular mechanisms of interval timing proposed here. In particular, the analysis of the signaling cascades coupled with mGluR7 in PCs is of great importance and should be a high priority. Since it is believed that cAMP, MAPK, and PI-3-K pathways are usually affected by mGluR7 activation, the contribution of each of them in temporal processing should be given a high priority for examination. Using immunoassay studies, region-specific changes in actin and microtubule dynamics should be monitored as a consequence of mGluR7 activation. Subsequently, the alterations in dendritic spines morphology should also be investigated. Additionally, the long-term effects of microtubule-stabilizing/destabilizing chemotherapy agents commonly given to cancer patients (e.g., paclitaxel and nocodazole) on PC’s firing patterns during eye-blink conditioning should be investigated in conjunction with the role of microtubules in the formation of intracellular temporal memories. Similar studies in the striatum would also be important for the establishment of the molecular mechanisms involved in both sub- and supra-second timing (Lusk et al., 2019).

The analysis of PC and MSN firing properties as a function of *what, whether, when*, and *how often* are becoming more amendable to study given the increase in the availability of online databases where the behavioral, pharmacological, and recording data can be reanalyzed in order to pursue new relations/interpretations while also trying to determine the value of placing older, less precise data in the context of new analysis tools that are rapidly becoming available. This will be especially important in the case of multiplexing the multiple lines of information contained in an individual neuron’s signal that is being combined with millions of other neuronal signals and propagated through various networks with the goal of determining time epochs of coincidence and extracting those data points of interest at specific nodes (e.g., Gallistel and King, 2010; Gu et al., 2015; Caruso et al., 2018).

Given the depth and breadth of the events and responses to be timed, as well as the vastness of the stimuli used and their diverse modalities, intensities, and patterns of presentation, it is not very surprising to discover that there is an extensive array of computational models of interval timing competing for our attention. Addyman et al. (2017) have been able to categorize competing models as a function of their core timing process, e.g., the speeds and types of variability of their pacemaker—accumulator, multiple oscillators, memory decay, climbing activations, random process and contextual change—with numerous implementations. Integrative concepts like the ICAT model, in particular, are only beginning to be investigated and understood at the neurobiological level as well as in practical, everyday terms (van Rijn, 2014; see Hartcher-O’Brien et al., 2016).

The SBF model of interval timing proposed by Matell and Meck (2000, 2004), attributes interval timing and temporal processing to the oscillatory input provided by cortical and thalamic projections to the striatal MSNs. Hence, corticostriatal synapses are responsible for encoding temporal memory in striato-thalamocortical circuits as described for a variety of different situations (e.g., Matell et al., 2003; Buhusi and Meck, 2005; Coull et al., 2011; Allman and Meck, 2012; and Merchant et al., 2013). It is also possible to consider the dynamic state of microtubules as an intracellular coincidence detector that regulates MSN spikes in accordance with the oscillatory patterns attained from cortical inputs (Buhusi et al., 2016). We have some insight into the contribution of BDNF in modulating glutamatergic and dopaminergic activity in cortico-striatal circuits, thereby regulating clock speed and the acquisition of timed response thresholds centered around a remembered target duration (e.g., MacDonald et al., 2012; Agostino et al., 2013; Lake and Meck, 2013). Moreover, histone deacetylase (HDAC) inhibition facilitates the acquisition of response thresholds in a peak-interval procedure through chromatin remodeling and microtubule stabilization (Yousefzadeh et al., 2018). Our current hypothesis is that all timing mechanisms are potentially regulated by coincidence detection at some level, i.e., molecular, cellular, or circuit levels (Agostino et al., 2011; Buhusi et al., 2016). Therefore, we propose that microtubule dynamics may be a viable candidate to serve as a fundamental constituent of temporal processing and information storage at the molecular level in both the cerebellar and striatal timing circuits. We also emphasize the need for conducting cell and molecular experiments to further elucidate the role of microtubule proteins in processing temporal and non-temporal information through synaptic plasticity-dependent or synaptic plasticity-independent manners.

## Author Contributions

All of the authors contributed equally to this hypothesis and theory article.

## Conflict of Interest

The authors declare that the research was conducted in the absence of any commercial or financial relationships that could be construed as a potential conflict of interest.
